# Risk of Stroke in Patients with Rheumatism: A Nationwide Longitudinal Population-based Study

**DOI:** 10.1038/srep05110

**Published:** 2014-06-05

**Authors:** Tsan-Hon Liou, Shih-Wei Huang, Jia-Wei Lin, Yu-Sheng Chang, Chin-Wen Wu, Hui-Wen Lin

**Affiliations:** 1 Department of Physical Medicine and Rehabilitation, Shuang Ho Hospital, Taipei Medical University, Taipei, Taiwan; 2 Graduate Institute of Injury Prevention and Control, Taipei Medical University, Taipei, Taiwan; 3 Department of Neurosurgery, Shuang Ho Hospital, Taipei Medical University, Taipei, Taiwan; 4 Graduate Institute of Clinical Medicine, Collage of Medicine, Taipei Medical University, Taipei, Taiwan; 5 Department of Rheumatology, Shuang Ho Hospital, Taipei Medical University, Taipei, Taiwan; 6 Department of Mathematics, Soochow University, Taipei, Taiwan; 7 Evidence-Based Medicine Center, Wan Fang Hospital, Taipei Medical University, Taipei, Taiwan; 8 These authors contributed equally to this work.

## Abstract

The aim of this study was to investigate rheumatoid arthritis (RA), and systemic lupus erythematous (SLE) as risk factors for stroke. The study was analyzed by Using the Taiwan Longitudinal Health Insurance Database 2005 (LHID2005), this cohort study investigated patients with a recorded diagnosis of RA (N = 6114), and SLE (N = 621) between January 1, 2004, and December 31, 2007, with age-matched controls (1:4) (for RA, N = 24456; SLE, N = 2484). We used Cox proportional-hazard regressions to evaluate the hazard ratios (HRs) after adjusting confounding factors. Our study found 383 of 6114 RA patients, experienced stroke during the 20267 person-year follow-up period. The adjusted HR of stroke for RA patients was 1.24 (95% CI, 1.11 to 1.39), and for SLE patients was 1.88 (95% CI, 1.08 to 3.27). When steroid was added as additional confounding factor, the adjusted HR of ischemic stroke for RA patients was 1.32 (95% CI, 1.15 to 1.50), and for SLE patients was 1.31 (95% CI, 0.51 to 3.34). In conclusion, the rheumatic diseases of RA, and SLE are all risk factors for stroke. After controlled the effect of steroid prescription, RA is risk factor for ischemic stroke.

Stroke patients are often left with neurological impairment. One-third to half of stroke patients have a major disability, and live lives of dependence on a daily basis^1^. Major symptoms include neurological impairment such as motor weakness, altered consciousness, sensory impairment, aphasia, and dysphagia. Stroke is a major cause of disability worldwide, and increases the economic burden on health care systems. Regarding the cost burden, stroke accounts for 2% to 4% of total healthcare costs worldwide, and in industrialized countries it accounts for more than 4% of direct healthcare costs^2^. Stroke is thus an important public health issue. Early risk detection is the primary means of stroke prevention.

In addition to well-known stroke risk factors such as hypertension, diabetes, hyperlipidemia obesity, and urbanization level^3^,^4^, some studies have shown that inflammation and chronic infection may also be risk factors^5^,^6^. Rheumatic diseases such as rheumatoid arthtitis (RA) and systemic lupus erythematous (SLE)result in chronic inflammation and increased risk of atherosclerosis, which is the most common pathologic process leading to cardiovascular disease (CVD), as well as myocardial infarction (MI) and stroke^7^. It is well documented that RA patients have an increased risk of cardiovascular disease^8^,^9^,^10^. Besides, a recent study revealed that RA patients relatively high incidence of cardiovascular events (such as cardiac death, acute coronary syndrome, symptomatic stroke, and congestive heart failure), despite of that they had few coronary risk factors^11^. However, analyses in previous studies of RA as a stroke risk factor has yielded inconsistent results^12^,^13^.

SLE is a chronic inflammatory disease involving all organ systems. It is well established that SLE induces accelerated atherosclerosis. Previous studies have indicated a higher annual incidence rate of cardiovascular events (6%–10%) among SLE patients compared with healthy people (1.5%)^14^,^15^,^16^. Besides, a population based study in Taiwan found SLE patients had higher hospital costs and 3 fold risk of stroke when comparing patients without SLE^17^. They also found younger SLE patients had much higher risk of stroke than old aged SLE patients. Recently, another population based study in United States demonstrated that about SLE patients had nearly doubled mortality and CVD event hazards compared to age-matched and sex-matched comparisons^18^. Although the SLE patients have higher risk of stroke had been investigated, the medication influence was not analyzed and further study is needed.

As mentioned above, studies have addressed the risk of stroke among patients with rheumatologic diseases. The relationship between rheumatologic disease and stroke has not yet been explored thoroughly. Studies that have investigated this relationship have revealed controversial results. Therefore, this study investigates rheumatologic diseases as risk factors for stroke with a nationwide population-based study.

## Methods

### Study population and study design

The Longitudinal Health Insurance Database 2005 (LHID2005), released by the Taiwan National Health Research Institutes, provided the database for our study. The database contains all the original claim data of 1000000 beneficiaries, including inpatient care, ambulatory care, dental care, prescription drugs, and ICD-9-CM (International Classification of Diseases, 9^th^ Revision, Clinical Modification) diagnostic codes. The beneficiaries were randomly sampled from 25.56 million persons in the Registry for Beneficiaries 2005,a system that covers almost 99% of the total population of Taiwan^19^. In the ethic aspect, the database used consisted of de-identified secondary data, the study met the requirements of the “Personal Information Protection Act” in Taiwan. The data were analyzed anonymously and the need for informed consent was waived approved by institution of review board.

The study cohort consisted of all patients who, according to the LHID2005, had been diagnosed autoimmune disease such as RA (ICD-9-CM codes 714.0), or systemic lupus erythematous (ICD-9-CM codes 710.0) between January 1, 2004, and December 31, 2007 (N = 6508, RA; N = 647, SLE). The ICD-9-CM was coded by a clinician specializing in rheumatology. The RA and SLE diagnoses are made according to diagnostic criteria stipulated by the American College of Rheumatology (ACR). For the purpose of a summarized diagnosis of RA and SLE, the 2 consecutive codings of RA and 15 consecutive SLE codings were incorporated in this study. Patients with missing variables such as date of birth and sex were excluded from the study (N = 72, RA; N = 18, SLE). Exclusion criteria included a diagnosis of stroke (ICD-9-CM codes 430–438) before that of RA, or SLE(N = 322, RA; N = 8, SLE).The resulting study cohort comprised 6114 RA, and 621 SLE patients.

We obtained control patients from the remaining patients in the LHID 2005 who had been registered between January 1, 2004, and December 31, 2007. Patients were excluded if they had been diagnosed as having RA, or SLE between 2004 and 2008 or with stroke before 2004. The selected control patients were matched with those in the study cohort (4 control patients per case patient) according to age (< = 30, 31–40, 41–50, 51–60, 61–70 and >70 y) and sex. All patients were observed from the date of cohort entry until they developed stroke or until the end of 2008.

### Baseline variables

We obtained baseline variables, including age, sex, urbanization level, diabetes mellitus (DM; ICD-9-CM codes 250), hypertension (ICD-9-CM codes 401–405), hyperlipidemia (ICD-9-CM codes 272.0–272.4), coronary heart disease (ICD-9-CM codes 410 and 412), congestive heart failure (ICD-9-CM codes 428, 398.91, and 402.x1), diabetes (ICD-9-CM codes250), hypercoagulability (ICD-9-CM codes289.81 and 286.9), renal disease (ICD-9-CM codes580–589), atrial fibrillation (ICD-9-CM codes427.31), and valvular heart disease (ICD-9-CM codes394–396, 424.0, and 424.1) for all patients. Urbanization levels were stratified into 3 levels. Regarding the urbanization level, all 359 cities or towns in Taiwan are stratified into 7 levels according to standards published by the Taiwanese NHRI, with Level 1 referring to “most urbanized” and Level 7 referring to “least urbanized.” For our study, Levels 1 and 2 were combined into a single group, and referred to as urban; Levels 3 and 4 were combined into a single group, and referred to as suburban; and the remaining 3 levels (5, 6, and 7) were combined into a single group, and referred to as rural. Steroid prescription coding, which is more than 30 days of prescription, was obtained for medication variant control in advanced step of analysis. Furthermore, other related medication such as aspirin, non-steroidal anti-inflammatory drugs (NSAIDs) and hydroxychloroquine were enrolled as confounding factors.

### Outcome measure

We identified the first diagnosis of stroke (ICD-9-CM codes 430–438) as the study end point. For stroke type analysis, we separated hemorrhagic stroke (ICD-9-CM codes 431) and compared the ischemic stroke in further adjusted hazard ratio analysis. All the study subjects were followed from the index date to occurrence of end point or until December 31, 2008, whichever was first, and the observations on the last dates were considered as censored observations.

### Statistical analysis

We compared demographic characteristics and co-medical disorders using Pearson's chi-squared test or Fisher's exact test. We used Cox models to evaluate hazard rates for stroke between the study cohort and control cohort after adjusting for potential confounding factors, including patient age (as continuous), sex, diabetes mellitus, coronary heart diseases, hypertension, hyperlipidemia, and urbanization level^20^. We checked each dichotomous variable in the model for proportionality by exploratory diagnostic log-log survival plots to meet the proportional-hazards assumption. We plotted the stroke hazard curves based on the Cox models for the patients and the control cohort after adjusting for potential confounding factors. For further analysis of medication related compounding factor analysis, steroid was taken as a variant for adjusted hazard ratio analysis among these two rheumatic diseases and different type of strokes. All analyses were performed using an SAS statistical package (SAS System for Windows, Version 9.1.3, SAS Institute Inc., Cary, NC, USA) and SPSS Version 20. A *P* value of <.05 was considered statistically significant.

## Results

**Figure 1:**
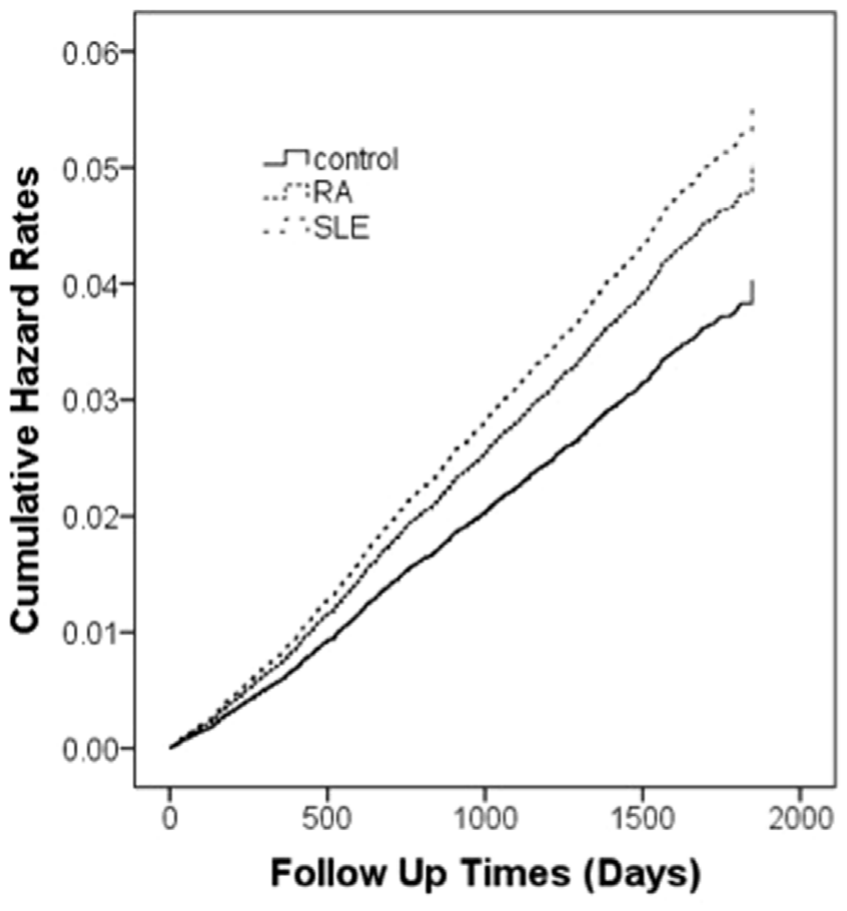
Plot of stroke hazard curves based on the Cox model analysis for patients with RA and SLE and comparison cohort after adjustment for age, sex, diabetes mellitus, hypertension, coronary heart diseases, hyperlipidemia, congestive heart failure, hypercoagulability, renal disease, atrial fibrillation, valvular heart disease, Aspirin use, Hydroxychloroquine use and NSAIDs use.

We identified 6114 RA, and 621 SLE patients and age- and sex-matched control cohorts. Compared to the control cohort in Table 1, RA patients were more likely to have comorbid hypertension (*P* < .001), hyperlipidemia (*P* < .001),coronary heart disease (*P* < .001), congestive heart failure (*P* < .01), renal disease (*P* <.001), and valvular heart disease (*P* < .001). SLE patients were more likely to have hypertension (*P* < .001), hypercoagulability disease (*P* < .01), renal disease (*P* < .001), and valvular heart disease (*P* < .05). Figure 1 shows the hazard curves for stroke in RA, and SLE patients and the control cohort during the 5-year follow-up period after adjustment for patient age, sex, diabetes mellitus, coronary heart disease, hypertension, hyperlipidemia, and urbanization level (log-rank test, *P* < .001).

Table 2 shows the incidence and hazard ratios for stroke in the RA, and SLE patients and the control cohort. Of the 6114 RA patients, 383 (a rate of 18.89 per 100000 person-years) experienced stroke during the 20267 person-year follow-up period. After adjustment for potential confounding factors, the adjusted HR of stroke(with both ischemic and hemorrhagic types) was1.24 (95% CI, 1.11 to 1.39), which was larger than those in the control group. Of the 621 SLE patients, 21 (a rate of 8.18 per 100000 person-years) experienced stroke during the 2567 person-year follow-up period, and the adjusted HR of stroke was 1.88 (95% CI, 1.08 to 3.27) after adjustment for potential confounding factors. The adjusted HR of stroke for the entire study cohort was 1.25 (95% CI, 1.12 to 1.40).

**Figure 2:**
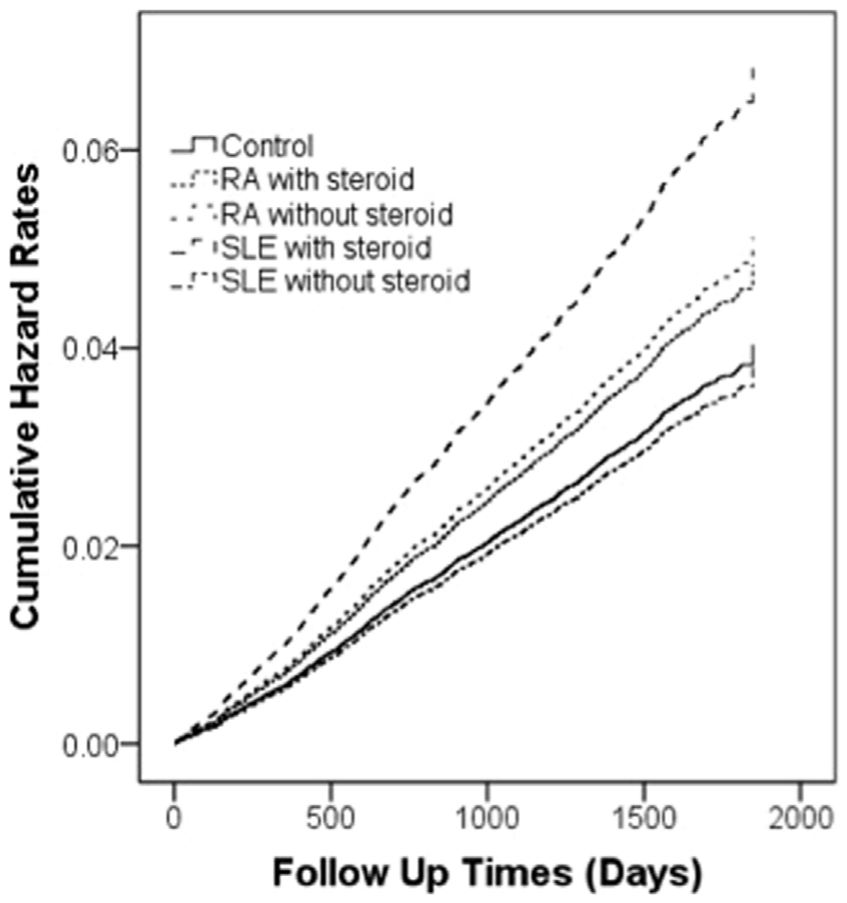
Plot of ischemic stroke hazard curves based on the Cox model analysis for patients with RA and SLE with or without steroid usage and comparison cohort after adjustment for age, sex, diabetes mellitus, hypertension, coronary heart diseases, hyperlipidemia, congestive heart failure, hypercoagulability, renal disease, atrial fibrillation, valvular heart disease, Aspirin use, Hydroxychloroquine use and NSAIDs use.

Table 3 shows the incidence and hazard ratios for ischemic stroke in the RA, and SLE patients and the control cohort. Furthermore, the steroid effect was taken as a confounding factor for ischemic stroke in this step of analysis. When steroid prescription was not enrolled in the confounding factor for ischemic stroke, all of the two rheumatic diseases are risk factors for ischemic stroke (adjusted HR: RA = 1.24,95% CI, 1.02 to 1.50, *P* < .05 and SLE = 2.04,95% CI, 1.07 to 3.91, *P* < .05)However, when steroid prescription was enrolled as confounding factor, RA (adjusted HR = 1.32, 95% CI, 1.15 to 1.50, *P* < .001) was risk factor for ischemic stroke. But there is no statistic significance of SLE as a risk factor for ischemic stroke. This may indicate that steroid could influence the developing of ischemic stroke for SLE patients. (Figure 2)When analyzing hemorrhagic type of stroke by adjusted HR analysis, There are no statistical significance of adjusted HR of RA(with steroid usage *P* = .600; without steroid usage *P* = .115), and SLE (with steroid usage *P* = .427; without steroid usage *P* = .994) patients.

## Discussion

Our study revealed that patients with autoimmune diseases such as RA, and SLE were more vulnerable to cerebral vascular accidents (CVA). And the analysis the risk of ischemic and hemorrhagic stroke types among these two rheumatic diseases revealed that there is no statistical significance of adjusted HR in hemorrhagic stroke. This can be explained that the pathogenesis theory of ischemic stroke and hemorrhagic stroke is different.

The pathogenesis of ischemic stroke in patients with autoimmune diseases, such as RA, and SLE, is described below. First, atherosclerosis is an immune-mediated inflammatory process^21^. The systemic inflammation in the pathogenesis of autoimmune diseases interacts and accelerates vessel atherosclerosis^22^. This mechanism is consistent with our observation that the incidence of stroke increased in patients who had suffered from autoimmune diseases for longer periods^23^. Second, endothelial dysfunction is a key pathologic process that occurs in early atherosclerosis. Endothelial function is impaired in systemic autoimmune diseases^24^,^25^. Third, traditional cardiovascular risk factors are more prevalent in patients with systemic autoimmune diseases^25^. Finally, medication for autoimmune diseases might play a role in the increased risk of atherosclerosis. NSAIDs are associated with increased cardiovascular risk and stroke^26^,^27^. Corticosteroids have been shown to be associated with higher cardiovascular risk, which might be a result of weight gain, adverse alterations in lipids, insulin resistance, and diabetes^28^.

Compared to studies for stroke risk of RA patients by Sodergren et al (odds ratio = 2.6) and Semb et al (odds ratio = 1.6), our study revealed a lower risk ratio (1.38)^12^,^13^. Other than ethnic differences, the lower risk may come from our study design with controlled confounding factors in the hazard ratio analysis. The systemic inflammation of RA has a direct effect on the endothelium, and predisposes patients to accelerated atherosclerosis^24^,^29^. Previous studies have shown that cytokines, chemokines, and autoantibodies oxidized low-density and matrix metalloproteinases (MMPs) are pathogenesis factors of atheroscelrosis^30^,^31^,^32^,^33^,^43^. In addition, smoking is a common risk factor for RA and stroke. Furthermore, RA can involve the heart valve structure and lead to atrial fibrillation, which is a well-documented risk factor for stroke^34^.

Our study revealed SLE as a risk factor for stroke, which is consistent with another population-based study conducted in Taiwan. SLE patients show a higher prevalence of traditional risk factors for atherosclerosis (i.e. hypertension, diabetes mellitus, and smoking) than the general population^16^. The associated anti-phospholipid syndrome can increase the risk of stroke^35^,^36^. In addition to the systemic inflammation process, lupus patients have been proven to have increased blood viscosity^37^, elevated homocysteinlevels^38^, and susceptible genetic background^39^,^40^. These factors have been shown to be associated with increased stroke risk. Furthermore, immunosupressants may be a factor for stroke. Leflunomide and cyclosporine may complicate hypertension^41^. However, these studies were only correlation studies, and did not prove causality. Further cohort studies are necessary to verify the effects of these factors. Therefore, we analyzed the effect of steroid usage in SLE patients for developing ischemic stroke. Our study found that when steroid was enrolled as a confounding factor of adjusted HR analysis, SLE was not a risk factor for ischemic stroke. This indicated that the steroid usage can lead the SLE patients more vulnerable to ischemic stroke. Doria et al found a correlation between cumulative steroid dosage and subclinical carotid atherosclerosis in lupus patients^42^. And the outcome of our study is compatible with previous study by Doria et al. SLE patients with steroid usage should be cautioned with ischemic stroke incidence and efforts for stroke prevention is recommended.

For better accuracy of ICD-9-CM diagnostic codes for stroke, we applied the stroke guideline for standard diagnosis and treatment. In Taiwan the Guideline for stroke has been setup for clinical application and Taiwan Stroke registry has been promoted by Taiwan Stroke Association since 2003. Besides, the accuracy of ICD-9 CM coding was checked by committees of the Bureau of the NHI that randomly sample the claim data and review the chart to verify the diagnostic validity regularly. The NHI claim database is a well established research database and has been used in various biomedical research fields. And the accuracy of the NHIRD (National Health Insurance Registration Database) in recording ischemic stroke diagnoses was high (97.85%), and the NHIRD appears to be a valid resource for population research in ischemic stroke. For the efforts of accurate register data acquirement, diagnosis of RA, and SLE were established by specialists in rheumatology based on clinical manifestations and laboratory data, which correlated with the American College of Rheumatology (ACR) 1997 revised criteria. In Taiwan, patients with these rheumatic diseases can apply for catastrophic illness registration cards from the Bureau of NHI and they do not need to additional copayment of using medical resources. For prevention of addition burden of medical insurance, the ICD9-CM coding for these rheumatic diseases is checked by the committees of the Bureau of NHI and the catastrophic illness registration card is allowed after the step of confirmation. In additional to confirmation by rheumatologist and the Bureau of NHI, we only enrolled consecutive coding cases for prevention of wrong coding in the database. These methods could improve the accuracy of these rheumatic diseases registration.

Nevertheless, this study is subject to several possible limitations. First, the diagnosis of SLE, RA, stroke, and medical comorbidities was entirely determined using the ICD codes from the National Health Insurance claim database and there may be concern about the diagnostic accuracy of the database. Even the accuracy of the National Health Insurance Registration Database (NHIRD) in recording ischemic stroke diagnoses was high (97.85%), and it appears to be a valid resource for population research in ischemic stroke^43^. However, there is limited information about the accuracy of RA, and SLE in NHIRD. Besides delayed coding could be occurred of the NHIRD and disease duration before this database is uncertain. These could influence the developing of strokes. Second, the NHIRD does not contain clinical data on the neurologic functional status of patients, or the location and size of cerebral infarct and hemorrhage. And the stratified of the rheumatic disease severity is limited in the NHIRD because the laboratory data and clinical scale cannot be represented. Third, the NHIRD lacks data on individual behaviors that are major risk factors for stroke such as smoking or alcohol consumption. It also does not contain the death records of enrollees. Absence of death records, however, could affect our study results if some of the deaths were stroke-related. Finally, to make precise diagnoses, we adopted various sampling methods in this study. This may result in the prevalence of these 2 rheumatic diseases not being represented collaboratively in this study.

## Conclusion

RA is risk factor for ischemic stroke in this population-based longitudinal cohort. And steroid is a crucial factor for contributing the risk of ischemic stroke among SLE patients. Due to the limited information can be obtained from our NHI database, further research is recommended with medication (such as DMARDs and immunosuppressive drugs), disease severity of stroke and rheumatic disease with more delicate study design in the future.

## Author Contributions

Conceived and designed the experiments: T.S.L., S.W.H., H.W.L. Performed the experiments: T.S.L., S.W.H., H.W.L. Analyzed the data: H.W.L. Prepare Tables and Figure: Y.S.C., J.W.L., C.W.W. Wrote the paper: T.S.L., S.W.H., Y.S.C. All authors reviewed the manuscript.

## Figures and Tables

**Table 1:** Baseline Variable for patients in the RA, and SLE cohorts and age- and sex-matched control cohorts

	RA patients n = 6114	Control patients n = 24 456	SLE patients n = 621	Control patients n = 2484
Baseline Variable	N (%)	N (%)	N (%)	N (%)
Age (years)				
≤30	515 (8.4)	2060 (8.4)	214 (34.5)	856 (34.5)
31–40	754 (12.3)	3016 (12.3)	152 (24.5)	608 (24.5)
41–50	1315 (21.5)	5260 (21.5)	132 (21.3)	528 (21.3)
51–60	1500 (24.5)	6000 (24.5)	66 (10.6)	264 (10.6)
61–70	1102 (18.0)	4408 (18.0)	40 (6.4)	160 (6.4)
>70	928 (20.0)	3712 (15.2)	17 (2.7)	68 (2.7)
Gender				
Male	1752 (28.7)	7008 (28.7)	69 (11.1)	276 (11.1)
Female	4362 (71.3)	17448 (71.3)	552 (88.9)	2208 (88.9)
Urbanization level				
Urban	3599 (58.9)	14289 (58.4)	383 (61.7)	1528 (61.5)
Suburban	1810 (29.6)	7160 (29.3)	167 (26.9)	696 (28.0)
Rural	705 (11.5)	3007 (12.3)	71 (11.4)	260 (10.5)
Diabetes mellitus				
Yes	2845 (9.3)	599 (9.8)	21 (3.4)	96 (3.9)
No	27725 (90.7)	5515 (90.2)	600 (96.6)	2388 (96.1)
Hypertension				
Yes	1397 (22.8)***	5017 (20.5)	95 (15.3)***	198 (8.0)
No	4717 (77.2)	19439 (79.5)	526 (84.7)	2286 (92.0)
Hyperlipidemia				
Yes	691 (11.3)***	2175 (8.9)	30 (4.8)	95 (3.8)
No	5423 (88.7)	22281 (91.1)	591 (95.2)	2389 (96.2)
Coronary heart disease				
Yes	548 (9.0)***	1769 (7.2)	28 (4.5)	79 (3.2)
No	5566 (91.0)	22687 (92.8)	593 (95.5)	2405 (96.8)
congestive heart failure				
Yes	726 (11.9)**	2569 (10.5)	27 (4.3)	101 (4.1)
No	5388 (88.1)	21887 (89.5)	594 (95.7)	2383 (95.9)
hypercoagulability				
Yes	7 (0.1)	13 (0.1)	3 (0.5)**	1 (0.0)
No	6107 (99.9)	24443 (99.9)	618 (99.5)	2483 (100.0)
renal disease				
Yes	298 (4.9)***	819 (3.3)	68 (11.0)***	37 (1.5)
No	5816 (95.1)	23637 (96.7)	553 (89.0)	2447 (98.5)
atrial fibrillation				
Yes	30 (0.5)	104 (0.4)	0 (0.0)	4 (0.2)
No	6084 (99.5)	24352 (99.6)	621 (100.0)	2480 (99.8)
valvular heart disease				
Yes	138 (2.3)***	337 (1.4)	12 (1.9)*	20 (0.8)
No	5976 (97.7)	24119 (98.6)	609 (98.1)	2464 (99.2)

Indicator *P < .05, **P < .01 and ***P < .001compared with the controls.

**Table 2:** Incidence, crude and adjusted hazard ratios (HRs), and 95% confidence intervals (CIs) for stroke in patients with RA, and SLE patients and in controls during a follow-up period of up to 5 years

Presence of stroke	Control	RA patients	*P* value
follow-up period			
Yes/Total	1490/24456	383/6114	
person-years	100636	20267	
Incidence per 100 000 person-years	14.80	18.89	
Crude HR (95% CI)	1.00	1.28 (1.14–1.44)	<.001
Adjusted HR^a^ (95% CI)	1.00	1.24 (1.11–1.39)	<.001
	Control	SLE patients	
Yes/Total	47/2484	21/621	
person-years	9379	2567	
Incidence per 100 000 person-years	5.01	8.18	
Crude HR (95% CI)	1.00	1.83 (1.08–3.08)	.024
Adjusted HR^a^ (95% CI)	1.00	1.88 (1.08–3.27)	.026
	Total controls	Total patients	
Crude HR (95% CI)	1.00	1.27 (1.14–1.42)	<.001
Adjusted HR^a^ (95% CI)	1.00	1.25 (1.12–1.40)	<.001

Notes:^a^Adjustments were made for age, sex, urbanization level, diabetes mellitus, hypertension, hyperlipidemia, coronary heart diseases, congestive heart failure, hypercoagulability, renal disease, atrial fibrillation, valvular heart disease, Aspirin use, Hydroxychloroquine use and NSAIDs use.

**Table 3:** crude and adjusted hazard ratios (HRs), and 95% confidence intervals (CIs) for ischemic stroke in patients with RA, SLE patients stratified by steroid use

Presence of ischemic stroke	Control	RA patients without steroid	RA patients with steroid
Crude HR (95% CI)	1.00	1.27*** (1.12–1.46)	1.46*** (1.21–1.77)
Adjusted HR^a^ (95% CI)	1.00	1.32*** (1.15–1.50)	1.24* (1.02–1.50)
		SLE patients without steroid	SLE patients with steroid
Crude HR (95% CI)	1.00	1.47 (0.62–3.46)	2.03* (1.11–3.87)
Adjusted HR^a^ (95% CI)	1.00	1.31 (0.51–3.34)	2.04* (1.07–3.91)

Notes: ^a^Adjustments were made for age, gender, urbanization level, diabetes mellitus, hypertension, hyperlipidemia, coronary heart diseases,congestive heart failure, hypercoagulability, renal disease, atrial fibrillation, valvular heart disease, Aspirin use, Hydroxychloroquine use and NSAIDs use.Indicator *p < 0·05, **p < 0·01 and ***p < 0·001compared with the control.
